# Essential Oils: Chemistry and Pharmacological Activities

**DOI:** 10.3390/biom13071144

**Published:** 2023-07-18

**Authors:** Damião P. de Sousa, Renan Oliveira S. Damasceno, Riccardo Amorati, Hatem A. Elshabrawy, Ricardo D. de Castro, Daniel P. Bezerra, Vitória Regina V. Nunes, Rebeca C. Gomes, Tamires C. Lima

**Affiliations:** 1 Department of Pharmaceutical Sciences, Federal University of Paraiba, João Pessoa 58051-900, Brazil; vitoriareginaventura@gmail.com (V.R.V.N.); rebecagomes123456@gmail.com (R.C.G.); 2 Department of Physiology and Pharmacology, Center of Biosciences, Federal University of Pernambuco, Recife 50670-901, Brazil; renan.oliveirasilva@ufpe.br; 3 Department of Chemistry “G. Ciamician”, University of Bologna, Via Gobetti 83, 40129 Bologna, Italy; riccardo.amorati@unibo.it; 4 Department of Molecular and Cellular Biology, College of Osteopathic Medicine, Sam Houston State University, Conroe, TX 77304, USA; hxe007@shsu.edu; 5 Department of Clinical and Social Dentistry, Federal University of Paraíba, João Pessoa 58051-970, Brazil; rcastro@ccs.ufpb.br; 6 Instituto Gonçalo Moniz, Fundação Oswaldo Cruz (IGM-FIOCRUZ/BA), Salvador 40296-710, Brazil; danielpbezerra@gmail.com; 7 Department of Pharmacy, Federal University of Sergipe, São Cristóvão 49100-000, Brazil; tamires.cl87@gmail.com

**Keywords:** natural products, metabolites, medicinal plants, volatiles, anti-inflammatory, antioxidant, antiviral, anticancer, antibacterial, antifungal

## Abstract

In this review, we provide an overview of the current understanding of the main mechanisms of pharmacological action of essential oils and their components in various biological systems. A brief introduction on essential oil chemistry is presented to better understand the relationship of chemical aspects with the bioactivity of these products. Next, the antioxidant, anti-inflammatory, antitumor, and antimicrobial activities are discussed. The mechanisms of action against various types of viruses are also addressed. The data show that the multiplicity of pharmacological properties of essential oils occurs due to the chemical diversity in their composition and their ability to interfere with biological processes at cellular and multicellular levels via interaction with various biological targets. Therefore, these natural products can be a promising source for the development of new drugs.

## 1. Introduction

The term essential oil was created in the 16th century and refers to the theory of “*Quinta essentia*” proposed by the famous German-Swiss alchemist and physician Paracelsus (1493–1541), born by Theophrastus Philippus Aureolus Bombastus von Hohenheim. Paracelsus defined the role of alchemy by developing plant extracts and herbal medicines. He believed that the distillation process extracted the most significant part of the plant, namely, the “*quintessence for healing*” or “*plant’s soul*”, separating the “essential” constituents from the “nonessential” [1,2].

Many authors have tried to supply a definition of essential oils. According to the “*Association Française de NORmalisation*” [3] and to the European Pharmacopoeia (Ph. Eur.), an “essential oil” can be defined as a “product obtained from a natural raw material of plant origin, either by distillation with water or steam, or from the epicarp of *Citrus* sp. fruits by a mechanical process, or by “dry distillation”. Essential oil is then separated from the aqueous phase by physical means” [3,4].

Plants can synthesize two kinds of oils: fixed and essential oils. Fixed oils are esters of a glycerol molecule attached to three fatty acids, also called triacylglycerols or triglycerides. Essential oils (EOs), also known as essences, volatile oils, etheric oils, or aetheroleum, are complex natural mixtures of volatile, lipophilic, and odoriferous substances commonly found in aromatic plants. The majority of essential oils are colorless or pale yellow, liquid at room temperature, and less dense than water, with very few exceptions (cinnamon, sassafras, and vetiver). Moreover, essential oil chemical constituents have a low molecular weight (below 300), and some of them are optically active, soluble in most organic solvents (ether, alcohol, acetone), and insoluble in water [2,5,6].

EOs have been widely investigated for their therapeutic potential in various pathologies [7,8,9]. Biological and pharmacological tests using EOs and their chemical constituents performed via experimental models at the molecular, cellular, and animal levels have generated promising results in several areas of science [10,11]. Their pharmacological profile includes antimicrobial, anti-inflammatory, antitumor, antioxidant activities, among others [10,12,13,14]. In view of this, in this review, relevant knowledge of the chemistry of essential oils is presented and their main mechanisms of action related to organic and infectious disorders are discussed.

## 2. Materials and Methods

The search was performed on PubMed/MEDLINE, and studies were selected according to their relevance and the objectives of this review. The following terms were used in combination within search strings: “anti-inflammatory”; “antioxidant”; “cancer”; “chemistry of essential oils”; “tumo(u)r”; “antibacterial”; “essential oils”; “antifungal”; “antimicrobial”; “antiviral”. The search was restricted to English and experimental studies.

## 3. Chemistry of Essential Oils

In general, essential oils are composed of approximately 20–60 components at different concentrations, but some of them may contain more than 300 different substances. However, two or three components are usually present in large proportions (20–70%) compared to other constituents present in small concentrations [15]. For example, rotundifolone is the major component (50–65%) of *Mentha* x *villosa* Hudson leaf essential oil [16], 1,8-cineole or eucalyptol (70–90%) of *Eucalyptus globulus* Labill. essential oil [17], and cinnamaldehyde (60–90%) of *Cinnamomum zeylanicum* Blume bark and leaf essential oil [18]. Typically, the major components of essential oils are the main components responsible for their biological properties. However, minor compounds may also play an important role in bioactivity, either by potentiating the action of major components or through antagonistic or additive effects [19].

Essential oil components possess distinct primary metabolic precursors and are generated through different biosynthetic pathways. They can be divided into two main groups: terpenoids (major group) and non-terpenoids (mainly phenylpropanoids). All of them are hydrocarbons and their oxygenated derivatives, and may exist in the form of several chemical classes, including aldehydes, ketones, alcohols, oxides, esters, amines, amides, phenols, nitrogen and sulfur compounds, and heterocycles [5,20,21,22].

### 3.1. Terpenes

Terpenes may be considered to be made up of isoprene units and constitute one of the largest and most structurally diverse families (>50,000 molecules) of natural products. Still more numerous than terpenes is a class of compounds named “terpenoids” (or isoprenoids). Terpenes are hydrocarbons, while terpenoids are a modified class of terpenes that present oxygen-containing functional groups, such as ketone, hydroxy, aldehyde, ether, or carboxylic moieties. Chemical structures of terpenes may range from linear to mono- or polycyclic compounds, and their skeleton is formed through the condensation of two to many thousands of 5-carbon-base (C5) units (isoprene units) [23,24,25].

Isopentenyl diphosphate (IPP) and its allylic isomer dimethylallyl diphosphate (DMAPP) are the universal precursors of isoprene molecules [26]. In higher plants, two distinct and independent biochemical routes are involved in isoprenoid biosynthesis: the mevalonate (MVA) pathway, the first and classically acknowledged route for biosynthesis of DMAPP and IPP, and the methylerythritol 4-phosphate (MEP) pathway (non-mevalonate pathway). Moreover, they may also proceed via a combination of both the MVA and the MEP routes. The MVA pathway is localized to the cytosol, whereas the MEP pathway is bound to the plastidic compartment [27,28].

**Figure 1:**
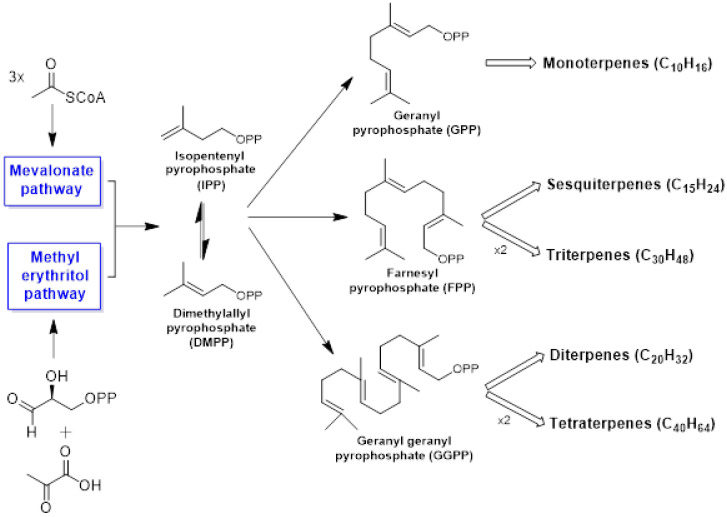
General scheme for the biosynthesis of terpenoids.

We can classify the terpenes into several categories according to the number of C5 building blocks in their core structure: hemiterpenes (C_5_H_8_), monoterpenes (C_10_H_16_), sesquiterpenes (C_15_H_24_), diterpenes (C_20_H_32_), triterpenes (C_30_H_48_), tetraterpenes or carotenoids (C_40_H_64_), and polyterpenes [(C_5_H_8_)_n_] [29]. Among the terpenic constituents, the mono- and sesquiterpenes are the most volatile and abundant in essential oils [30,31]. Figure 1 contains a general scheme for terpenoid biosynthesis.

**Figure 2:**
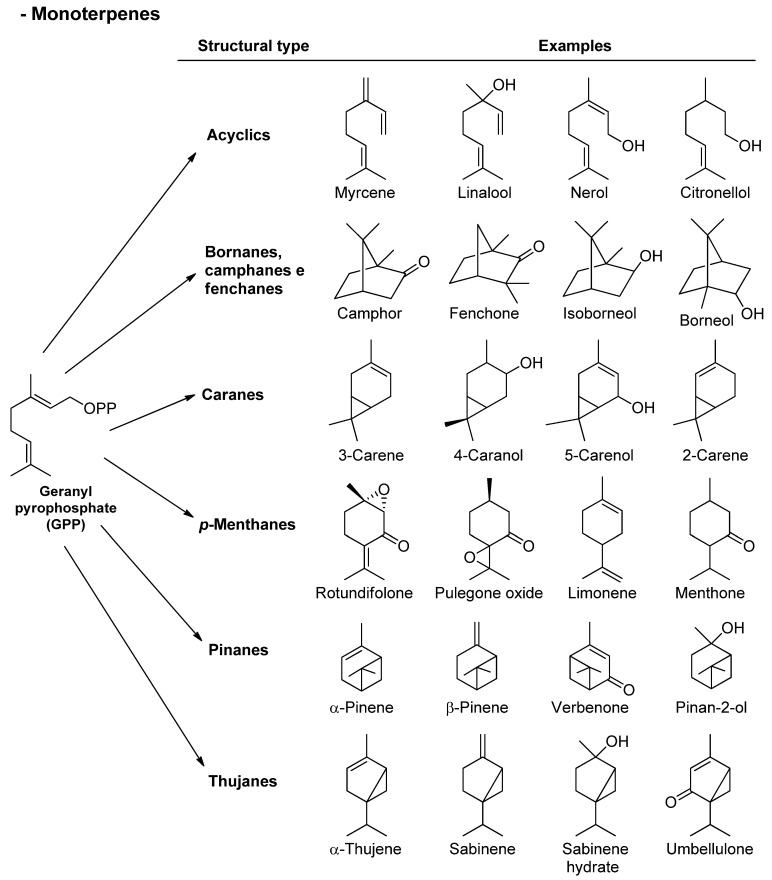
Chemical structures of monoterpenes found in essential oils.

Monoterpenes (from the Latin mono, or one) are the most representative and simple terpenes. They are produced from the assembly of two isoprene units (C10), constituting about 90% of plant essential oils and permitting a very large variety of chemical structures (around 1000 metabolites). According to their structural variation, monoterpenes can be divided into different subgroups: acyclics, bornanes, camphanes and isocamphanes, fenchanes, thujanes, *p*-menthanes, and caranes and pinanes (Figure 2). The variety of possibilities in which these basic skeletons can be rearranged results in amazing structural diversity observed in nature, in which *p*-menthane-type monoterpenes are the largest group of naturally occurring monoterpenes [21,32].

**Figure 3:**
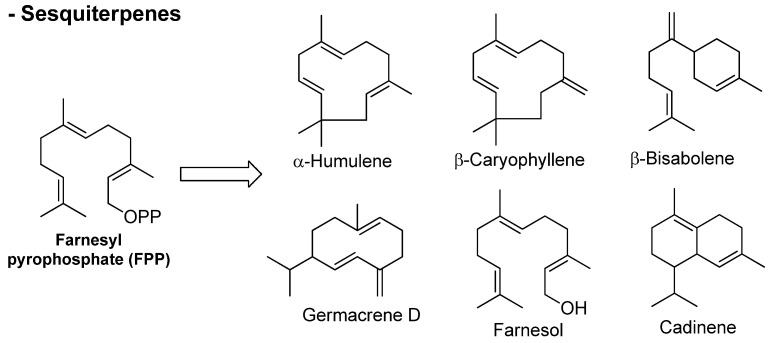
Chemical structures of sesquiterpenes found in essential oils.

Sesquiterpenes (from the Latin *sesqui*, or one and a half) are built from the coupling of three isoprene molecules (C15), occurring naturally in insects and higher plants. They are the most diverse group of terpenoids and may have acyclic (linear), monocyclic, bicyclic, or tricyclic frameworks, showing many unique arrangements. Similar to monoterpenes, sesquiterpenes can also occur as hydrocarbons or contain oxygen functionality, including carboxylic acids, lactones, alcohols, aldehydes, ketones, and epoxides. Chain extension with augmentation in a number of cyclizations as well as biochemical modifications (rearrangement or oxidation) allows a good variety of structures from the sesquiterpenes (Figure 3) [21,33].

### 3.2. Phenylpropanoids

**Figure 4:**
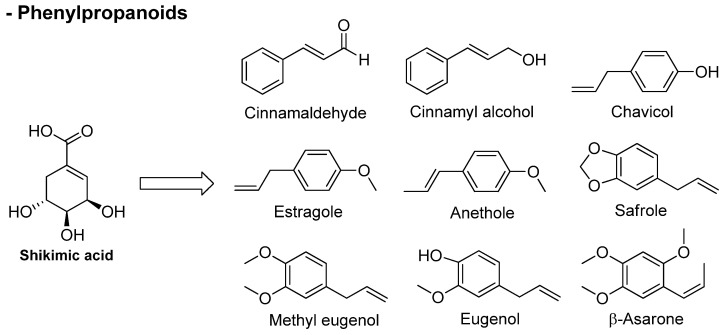
Chemical structures of phenylpropanoids found in essential oils.

Phenylpropanoids constitute a group of organic compounds synthesized by plants via the shikimate pathway; the aromatic amino acid L-phenylalanine is their biogenetic precursor. The core of phenylpropanoids consists of a phenyl ring connected to a C3 propane moiety. They are found in the plant kingdom, but are less common than terpenes. The wonderful diversity of phenylpropanoids derives from the efficient modification of a very limited set of core structures. Aromatic compounds originated from the shikimate route (Figure 4) comprise aldehyde, phenol, alcohol, methoxy, and methylenedioxy compounds [34,35]. Chemical structures of different essential oil constituents (mono- and sesquiterpenes, and phenylpropanoids) are depicted in Figure 2, Figure 3 and Figure 4.

Generally, essential oils represent a small fraction of plant composition (less than 5% of vegetal dry matter), occurring in specialized secretory structures, such as secretory ducts or cavities, glandular trichomes, and oil cells [36,37]. Volatile oils can be extracted from seeds (*Foeniculum vulgare* Mill.), flowers (*Rosa abietina* Gren. ex H. Christ), leaves (*Mentha* × *piperita* L.), barks (*Cinnamomum cassia* (L.) J. Presl), fruits (*Citrus sinensis* (L.) Osbeck; *Spondias mombin* L.), grasses (*Cymbopogon citratus* (DC.) Stapf.), tree blossoms (*Cananga odorata* (Lam.) Hook. f. & Thomson), rhizomes (*Zingiber officinale* Roscoe), roots (*Vetiveria zizanoides* (L.) Nash), woods (*Juniperus virginiana* L.), gums (*Boswellia ameero* Balf. f.), and bulbs (*Allium sativum* L.) [38,39]. Typically, essential oils obtained from different plant organs of the same plant possess specific chemical compositions.

Volatile oils are reasonably widespread in the plant kingdom, being rarely found in both gymnosperms and monocotyledonous angiosperms, and widely distributed in dicotyledonous angiosperms [38]. They are synthesized by more than 17,500 vegetal species, belonging to several genera distributed in approximately 60 families. Some plant families are well-known for their ability to produce aromatic plants rich in essential oils, including Apiaceae (Umbelliferae), Hypericaceae, Myrtaceae, Poaceae, Asteraceae (Compositae), Lamiaceae (Labiatae), Zingiberaceae, Cupressaceae, Lauraceae, Pinaceae, Piperaceae, and Rutaceae [40]. All of the essential oils-producing plant families are rich in terpenoids, while phenylpropanoids are more commonly found in specific plant families, including Apiaceae, Lamiaceae, Myrtaceae, Piperaceae, and Rutaceae [41].

The chemical composition and quality of essential oils may vary due to various factors, such as the number and type of molecules, stereochemistry of constituents, and employed extraction process (hydrodistillation, effleurages, Soxhlet extraction, cold pressing, supercritical fluid extraction). Furthermore, environmental conditions (climate, cultivation, plant organ, harvesting time, light intensity, soil composition, age, and vegetative cycle stage extraction) may also lead to changes in the quality, quantity, and composition of extraction products [42].

Currently, about 3000 aromatic plants are known to produce essential oils as an important part of their secondary metabolism, 300 of which are commercially significant due to their medicinal and industrial values. They are employed in different applications, such as pharmaceuticals, agronomy, sanitary applications, cosmetics, perfumes, dentistry, agriculture, and food [24,43].

## 4. Antioxidants

### 4.1. Mechanism of Antioxidant Activity of Main Essential Oils and Their Constituents

**Scheme 1:**
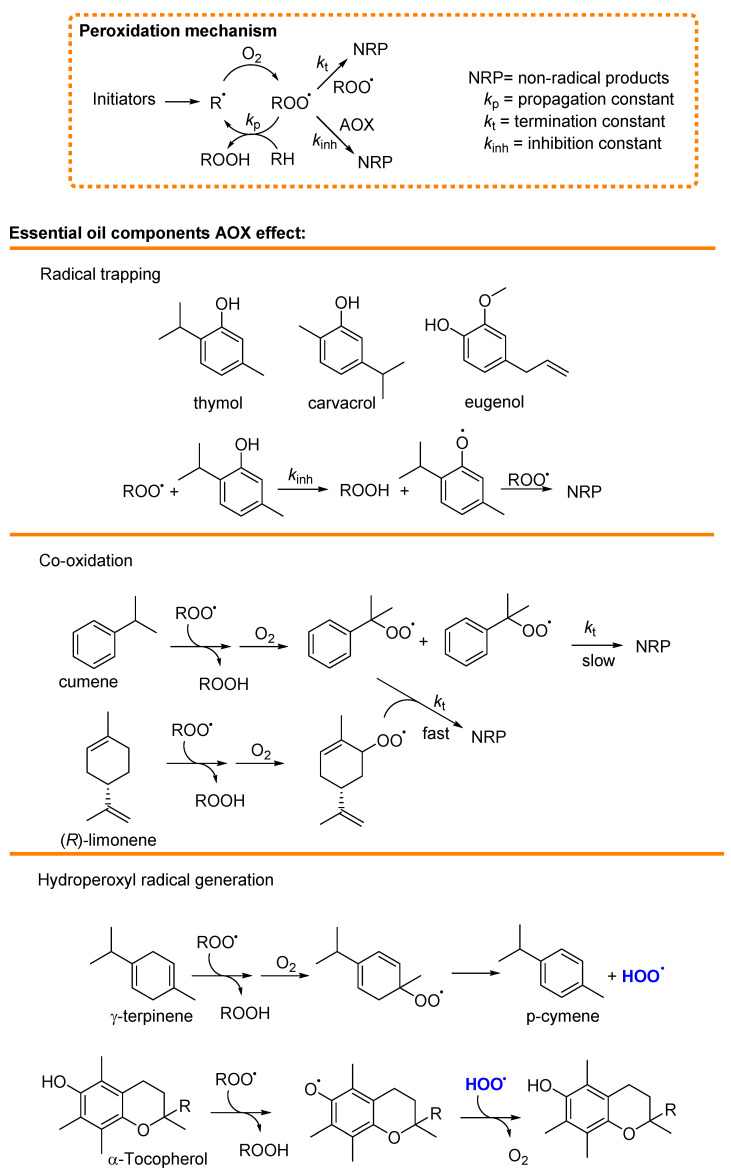
Chemical mechanism explaining the ability of essential oil components to retard the peroxidation of organic compounds (RH). The two-faced reducing and oxidizing hydroperoxyl radical is evidenced in blue.

An essential oil is a complex mixture of components, each of which can potentially contribute to its antioxidant activity. As the composition can be influenced by many factors, knowing the efficacy and the mechanism of action of each component allows us to predict the activity of the oil [44]. From a chemical point of view, antioxidant activity is defined as the ability of a given compound, present in small amounts, to protect an easily oxidizable material, such as polyunsaturated lipids, from oxidation [45]. Peroxidation is a radical-chain reaction responsible for the incorporation of O_2_ into organic molecules, leading to the formation of hydroperoxides, epoxides, and other oxygenated derivatives (Scheme 1). The main chain-carrying radicals of peroxidation are alkylperoxyls (ROO^•^), although other short-lived radicals can play a role in initiation (such as HO^•^, formed by the Fenton reaction) or in propagation (R^•^ and RO^•^) [45]. The antioxidant activity is, therefore, deeply related to the ability of a given molecule to trap ROO^•^ radicals and is described by two independent parameters: the number of radicals trapped by the antioxidant (also known as “capacity” or stoichiometric coefficient) and the rate constant of the reaction with ROO^•^ radicals, *k*_inh_. With respect to the chemical mechanism of peroxidation inhibition, essential oil components can be divided into three main groups: phenols, which act as radical trapping agents; highly oxidizable compounds, which enhance the termination; and 1,4-cyclohexadienes, which are able to generate the reducing HOO^•^ radical.

#### 4.1.1. Radical Trapping

Phenolic components comprise mainly carvacrol, thymol, and eugenol. Their antioxidant activity is due to the well-known ability of phenols to donate the phenolic H-atom to ROO^•^ radicals to form resonance-stabilized phenoxyl radicals unable to propagate the oxidative chain (Scheme 1). A recent study has shown that the *k*_inh_ and the stoichiometry of ROO^•^ trapping of phenolic containing essential oils is equal to the sum of the contributions of their phenolic components [46].

#### 4.1.2. Termination Enhancers

**Figure 5:**
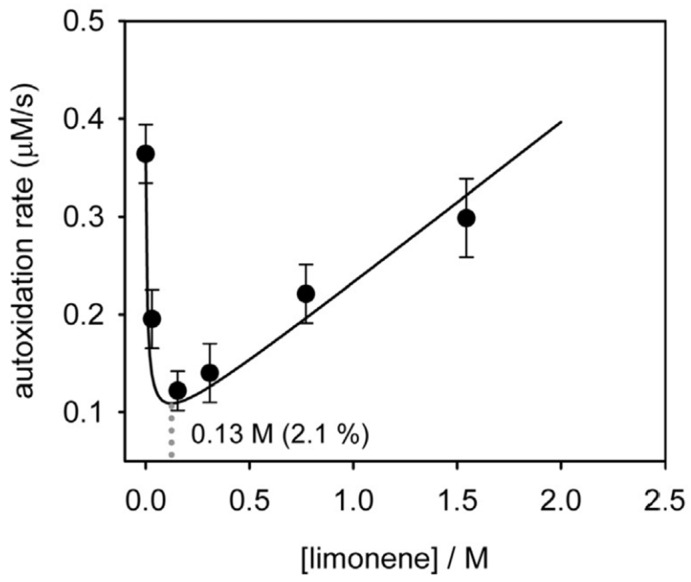
Rate of O_2_ uptake during the autoxidation of cumene (3.5 M) in chlorobenzene initiated by AIBN (0.05 M) at 30 °C as a function of the concentration of limonene. The concentration of limonene (in M and %, *v/v*) affording the strongest inhibition is indicated by the grey dots. Reprinted from Reference [48] with permission from Elsevier.

In specific cases, small quantities of a highly oxidizable essential oil component can slow down the autoxidation of another oxidizable substrate. The overall effect is strictly related to the nature of the substrate to be protected, as it must have a small propagation constant (*k*_p_) associated with a low termination constant (*k*_t_). Typical values of *k*_p_ and *k*_t_ of the oxidizable substrate are available in the literature [47]. Highly oxidizable essential oil components are oxidized with the substrate and, if they form peroxyl radicals able to decay with a high *k*_t_, they cause an overall decrease in the ROO^•^ concentration (Scheme 1). An example of this mechanism has been reported by using cumene as an oxidizable substrate, that has low values of *k*_p_ and *k*_t_. The diagram of cumene autoxidation rate vs. essential oil component concentration displays a typical “v” shape because, above a critical concentration, the autoxidation of the essential oil component becomes prevalent (see Figure 5) [48]. In principle, this mechanism could apply to oxidizable substrates in micellar or emulsion systems, where *k*_p_ and *k*_t_ are low because they are reduced by diffusion processes. Highly oxidizable essential oil components, as they are small molecules, can diffuse more easily, contributing to the decay of ROO^•^ radicals. This mechanism may account for the antioxidant activity of essential oils having no obvious radical trapping components.

#### 4.1.3. Hydroperoxyl Radical (HOO^•^) Generation

Among the highly oxidizable components, γ-terpinene (γ-T) has a unique behavior, leading to an unusual antioxidant activity. After H-atom abstraction, γ-T forms an unstable peroxyl radical which breaks down, yielding *para*-cymene and a hydroperoxyl (HOO^•^) radical (see Scheme 1). This radical has both oxidizing and reducing activity; therefore, it can donate a H-atom with very high rate constant to ROO^•^, to the radicals of an antioxidant [49], or to mild oxidizers such as *ortho*- and *para*-quinones [50]. γ-Terpinene was shown to reduce the rate of autoxidation of methyl linoleate [51,52] and to prolong the duration of the antioxidant activity of α-tocopherol and polyphenols at low (30 °C) [53] and high (130 °C) [54] temperatures. In addition, γ-T enables the antioxidant activity of melanins, which are natural polymers rich in *ortho*-quinone residues [50].

### 4.2. Methods Suggested to Explore the Radical Trapping Activity

The antioxidant activity of essential oil components is deeply connected to the nature and the physical state of the oxidizable substrate and to their interaction with ROO^•^ radicals. These pieces of information can hardly be obtained by using simplified assays based on the decay of stable radicals, or without the presence of an oxidizable substrate. The use of assays such as DPPH, ABTS, FRAP, and ORAC is, therefore, discouraged, despite the appeal of their simplicity and low cost [45]. More significant insights can instead be achieved by using methods relying on the autoxidation of biologically relevant substrates, such as polyunsaturated lipids (i.e., methyl linoleate, unsaturated phospholipids) in different aggregation states (solution, micelles, vesicles). The oxidation can be accelerated by using azo initiators, iron salts with peroxides, or simply by heating. Recommended methods to measure the progress of the reaction are determination of hydroperoxides or conjugated dienes, formation of malondialdehyde (thiobarbituric reactive substances, TBARS assay), O_2_ consumption, β-carotene bleaching test, or consumption of fluorescent probes of lipid peroxidation such as STY-BODIPY [45]. Examples of determination of the antioxidant activity of selected essential oils are reported in Table 1. 

## 5. Antiviral Activity

Essential oils are active against multiple DNA and RNA viruses, including herpes simplex virus type-1 (HSV-1) and type-2 (HSV-2), poliovirus, adenovirus, dengue virus type-2, yellow fever virus, influenza virus, respiratory syncytial virus, Zika virus, coronaviruses, coxsackievirus B-1, and Junin virus [58,59,60,61]. 

Oregano and clove EOs demonstrated potent antiviral activities against adenovirus, coxsackievirus B-1, and poliovirus [62]. *Melaleuca alternifolia* Cheel. (tea tree) EO showed in vivo antiviral activity against Tobacco Mosaic Virus (TMV) [63]. Several reports have demonstrated the antiviral activity of tea tree, manuka, eucalyptus, and thyme EOs against HSV-1 due to their contents of monoterpenes, phenylpropanes, and sesquiterpenes [64,65,66,67]. *Melaleuca alternifolia* essential oil and some of its components, such as terpinen-4-ol, terpinolene, and α-terpineol, inhibited the replication of influenza A/PR/8 virus subtype H1N1 [68]. The mechanism of antiviral activity appears to be due to inhibition of viral replication. Another study has shown that EOs of *Glechon spathulata* Benth., *Artemisia arborescens* L. and *Glechon marifolia* Benth. possess antiviral activities against HSV-1 [69,70], whereas *Melissa officinalis* L. EO inhibits the replication of HSV-2 [58]. *Eupatorium patens* D.Don ex Hook. & Arn. and *Artemisia douglasiana* Besser. EOs demonstrated antiviral activity against the dengue virus [68]. Interestingly, Pogostenon cablin and lemon balm EOs were reported for their antiviral activities against influenza A H2N2 virus and H9N2, respectively [71,72]. Moreover, lemon balm EO inhibited influenza H9N2 replication in a dose-dependent manner and the inhibition was enhanced by seltamivir [72]. 

The antiviral activities of essential oils have been attributed to the ability of essential oils and their components to interfere with viral entry through viral envelope disruption, capsid disintegration, or inhibition of viral binding to host cell receptors. Moreover, the antiviral activities of essential oils could be due to the inhibition of viral replication.

Alterations of viral particles (virus envelope disruption or capsid disintegration) have been described as antiviral mechanisms of essential oils. Gilling et al. reported that 4% oregano essential oil expanded the viral particle size of murine norovirus [73]. Moreover, treatment with 0.5% carvacrol resulted in the expansion of murine norovirus viral particles, capsid disintegration, and loss of viral infectivity [73]. 

Garozzo et al. showed that tea tree EO and its major constituent terpinen-4-ol impaired endolysosomal compartment acidification, which inhibited influenza viral uncoating and entry into target cells [74]. Marjoram, clary sage, *Thymus vulgaris* L., *Cinnamomum zeylanicum* Blume., *Citrus bergamia* Risso & Poit., and anise EOs have also been shown to inhibit influenza virus with a half maximal inhibitory concentration (IC50) < 100 μg/mL [75,76]. It was reported that curcuma EO constituent germacrone demonstrated high efficacy against influenza virus with IC50 of 6.03 μM and selectivity index (SI) of >41 [77]. Germacrone was also shown to inhibit not only the influenza virus but also feline caliciviruses [78]. Moreover, carvacrol, eugenol, and β-santalol have demonstrated potent anti-influenza virus activities [74,79], including *Cinnamomum zeylanicum* Blume. essential oil [80]. 

Feriotto et al. demonstrated that EOs of *Thymus vulgaris* L., *Cymbopogon citratus* (DC.) Stapf, and *Rosmarinus officinalis* L. bound to Tat protein of HIV and destabilized the Tat/TAR-RNA complex, which is required for HIV replication, at IC50s of 0.05–0.83 μg/mL [81]. The antiviral activity of thymol, carvacrol and other components of these essential oils has been reported [76,82,83]. Therefore, it is suggested that these components can contribute to the inhibitory action of essential oils that contain them against different viruses. Another study found that *Cymbopogon nardus* (L.) Rendle. EO inhibited HIV-1 reverse transcriptase with an IC50 of 1.2 mg/mL, and the activity was attributed to β-citronellol [84]. 

Significant numbers of studies have shown that EOs from Star Anise, Australian tea tree, oregano, *Eucalyptus caesia* Benth., and *Mentha suaveolens* Ehrh. exhibit antiviral activities against HSV-1 due to adsorption on viral particles and inhibition of viral attachment to host cells [65,76,85]. Star anise EO was found to be the most potent, with an IC50 of 1 μg/mL and a selectivity index (SI) of 160 [76]. Analysis of EOs’ active ingredients identified thymol and carvacrol with antiviral activity (IC50 of 7 µM) and β-caryophyllene (IC50 of 0.25 μg/mL and SI of 140) as the most active anti-HSV-1 components [86,87]. The inhibitory activity of β-caryophyllene was also reported against the dengue-2 virus, with an IC50 of 22.5 μM and SI of 71.1 [76]. In fact, studies have shown anti-HSV-1 activities also of eugenol, limonene, β-pinene, farnesol, and *p*-cymene [86,87,88,89]. These findings indicate that essential oils from plants containing appreciable amounts of these components, such as β-caryophyllene, could exhibit antiviral activity, including against HSV-1.

EOs have been shown to be effective not only against enveloped viruses but also against non-enveloped viruses. An in silico study suggested that *Lavandula stoechas* L. essential oil could inhibit SARS-CoV-2 [90]. *Osmunda regalis* L. EO showed anti-coxsackie viral activity, with an IC50 of 2.24 μg/mL and SI of 789.84, which indicate its high efficacy and low toxicity [76]. It has been reported by another group that oregano EO and its major component, carvacrol, is an effective antiviral against human rotavirus [91]. The previous data (Table 2) indicate that EOs and their constituents, either individually or in combination, may serve as potential antivirals against several viral pathogens.

## 6. Antimicrobial Activity 

Essential oils are recognized for their role in protecting plant structures against microorganisms. This supports the scientific hypothesis that such oils and their constituents may also present antimicrobial effects against pathogens of human interest. Most scientific investigations to evaluate the antimicrobial effects of essential oils and their constituents are conducted to determine the presence or absence of pharmacological effects and the lowest concentrations capable of inhibiting microbial growth. Research techniques, in general, seek to standardize methodological procedures and define breakpoints, and are thus recommended by research institutions, such as the Clinical and Laboratory Standards Institute (CLSI) and the European Committee on Antimicrobial Susceptibility Testing (EUCAST), which, in many countries, are in turn recognized by regulatory agencies for drug registration. Generally, the techniques include the adoption of agar diffusion and dilution methods [95]. 

Pharmacological knowledge of antimicrobial activity must involve elucidating the mechanism of action of the evaluated oil and providing information concerning possible toxicological effects and drug interactions. In addition, knowledge of the molecular aspects involved in the observed effects helps to advance proposals involving possible structural changes (to discover new molecules). In this review, we highlight the main mechanisms of action of essential oils and their constituents, which involve their antibacterial, antifungal, antiviral, and antiparasitic activities.

### 6.1. Antibacterial Activity

Although different microbial species represent different molecular targets, the modes of action of substances with antimicrobial activity generally involve functional components of the plasma membrane, the cell wall, cell duplication, or protein synthesis [96]. 

Observing the effect of essential oils on bacterial morphology helps to understand possible mechanisms of action. Microscopic visualization of changes in the cell wall, the membrane, or cell shape, revealed that the essential oil of *Citrus medica* L. promoted action against *Escherichia coli* and *Staphylococcus aureus*, with cell wrinkling, formation of holes in the bacterial surface, or even plasma membrane ruptures [97]. Essential oils from *Origanum* species showed antibacterial activity against *Haemophilus influenzae* and *Haemophilus parainfluenzae*. Scanning electron microscopy analyses of the *H. influenzae* and *H. parainfluenzae* biofilms showed a decrease in the appearance of bacterial clusters due to the action of *O. majorana* L. essential oil [98].

Due to hydrophobicity, EOs likely act on lipids of the bacterial plasma membrane or mitochondria, functionally impairing these structures, by promoting increased proton permeability, which is measured through membrane electrical conductivity tests [99]. The essential oil of mustard showed damage to bacterial cell membranes of *E. coli* and *Salmonella typhi* with loss of cellular structures essential for the survival of the microorganism, through the occurrence of ATP depletion and decreased intracellular pH [100]. The essential oil of *Origanum compactum* Benth. is capable of inducing the dissipation of gradients of potassium ions, causing loss of action potential in the membrane of *Pseudomonas aeruginosa* [101]. Observations from scanning electron microscopy indicated that the essential oil of *O. compactum* Benth. promoted cell wall damage in *E. coli* and *Bacillus subtilis* [102]. The rupture of the membrane and cell wall is directly related to the decrease in ATP production at the cell membrane. Carvacrol and thymol promote a decrease in the amount of intracellular ATP and increase extracellular ATP in *E. coli*. This effect indicates a disrupter on the cytoplasmic membrane [103]. 

Additionally, the chemical diversity in the composition of essential oils increases the possibility of existing constituents that can impair the synthesis of proteins. Analysis by sodium dodecyl sulfate–polyacrylamide gel electrophoresis confirmed by Western blotting indicated that carvacrol and *p*-cymene can promote a decrease in protein synthesis in *E. coli* [104]. Essential oils may also show mutagenic activity [105]. This effect can be analyzed in gene modulation studies [106,107]. The essential oil of *Syzygium aromaticum* (L.) Merrill & Perry showed action on the formation of bacterial biofilms, affecting the quorum sensing activity [108]. 

### 6.2. Antifungal Activity

Essential oils and their constituents act on fungal cell structures in a manner similar to that described for their antibacterial activity: the oils cause changes in functions that are essential for microbial survival. The main targets for these substances are involved in the maintenance of the fungal membrane and cell wall.

Changes in the permeability of the plasma membrane are largely related to the effects of essential oils involving ergosterol. The most studied mechanisms of action include both ergosterol biosynthesis modulation and direct ergosterol binding; these effects were visualized for essential oils of *Anethum graveolens* L. and *Coriandrum sativum* L., respectively [109,110]. It has not yet been fully elucidated, but the enzymes involved in the formation of ergosterol can also function as pharmacological targets. 

The effect of essential oils of *Anethum graveolens* L. on the fungal cell membrane promotes an increase in proton pumping activity, with consequent acidification induced by the presence of glucose in the external environment. The action on the mitochondrial membrane may contribute to decreasing intracellular ATPase activity, possibly by action on mitochondrial dehydrogenases. This cellular damage can be measured by the increased production of reactive oxygen species (ROS), an important biochemical marker for the apoptosis process [111,112].

Inhibiting the formation of the fungal cell wall is another potential mechanism of action of essential oils and their constituents. In vitro experimental models, supplemented with sorbitol (an osmotic protector) in the culture medium, and in silico computational studies indicate that certain oils can act on enzymes such as delta-14-sterol reductase and 1,3-β-glucan synthase, which are involved in cell wall synthesis [113,114].

Evaluation of the effect of essential oils and their constituents on fungal micromorphology has helped augment our knowledge of their mechanisms of action. The essential oil of *C. sativum* caused a significant reduction in the development of hyphae, pseudohyphae, chlamydoconidia, and blastoconidia in *Candida albicans* [109]. The essential oil of *C. sativum* L. commonly has a significant amount of linalool and a smaller amount of *gamma*-terpinene in its chemical composition [112]. These monoterpenes have antifungal action against various strains of fungi [115,116]. Therefore, they may contribute to the antifungal activity of essential oils that contain them. Table 3 presents the principal targets involved in the activity of essential oils and their constituents against fungi and bacteria.

## 7. Antitumor Activity

The in vitro cytotoxic and in vivo antitumor effects of EOs have been widely reported. In particular, many mechanisms of action have been proposed. These include induction of apoptotic cell death that was related to the increase in the level of reactive oxygen species (ROS), suppression of the AKT/mTOR and NF-κB pathways, and activation of the MAPK pathway. For example, EO from the bulb of *Allium sativum* L. caused cytotoxicity in leukemia cells through the increase in ROS and induction of apoptosis and differentiation [118], while *Zataria multiflora* Boiss. EO increased ROS and induced apoptosis in colon cancer cells [119]. The EO from seeds of *Litsea cubeba* (Lour.) Pers. induced apoptotic cell death by suppressing the AKT/mTOR pathway [120]. On the other hand, the cytotoxicity of *Cedrus deodara* (Roxb. ex D. Don) G. Don bark EO [121] and *Euphorbia intisy* Drake stem EO [122] was related to the inhibition of the NF-κB pathway. Moreover, *Artemisia capillaris* Thunb. EO induced apoptotic cell death by activating MAPK [123]. These mechanisms of cytotoxic action are related to the chemical composition of each EO. For example, the in vivo studies carried out on perillyl alcohol showed that this monoterpene can inhibit the prenylation of specific proteins by type I and type II geranylgeranyl-protein transferases. However, it has been shown that it does not alter the farnesyl-protein transferase enzyme in NIH3T3 cells [124]. In addition, the cytotoxic action of perillyl alcohol was investigated against pancreatic cancer cells. It is suggested to inhibit the prenylation of growth-regulatory proteins, such as K-Ras and H-Ras [125]. Furthermore, studies on thymoquinone, an essential oil component found in the *Nigella sativa* L. plant, showed that it induces p53-independent apoptosis, as reported in tests using human osteosarcoma cells [126]. *Psidium guajava* L. leaf essential oil showed significant cytotoxic activity against human oral epidermal carcinoma. The main chemical constituents of this oil are limonene (38.01%) and β-caryophyllene (27.98%) [127]. Limonene has antitumor action via several mechanisms of action [13,128,129]. For example, it induces apoptosis of lung cancer cells by promoting autophagy in lung cancer cells [130]. The antitumor action of β-caryophyllene has also been reported. This compound enhances the antitumor activity of cisplatin in lung cancer cell lines via regulation of the cell cycle and apoptosis signaling molecules [131], in addition to other mechanisms of action in several tumor cell lines [128,129]. Therefore, limonene and β-caryophyllene may contribute to the antitumor activity of *Psidium guajava* L. leaf essential oil and other antitumor oils in which these constituents are present in significant amounts. Table 4 summarizes some cytotoxic/antitumor mechanisms related to EOs. 

## 8. Anti-Inflammatory Activity 

### 8.1. Inflammatory Response

Inflammation is a complex protective response against harmful exogenous stimuli or endogenous signaling, where the immune system plays a key role in eliminating the initial cause and preserving the cellular and tissue structure, which culminates in maintaining homeostasis [147]. The persistence of this response in the absence of aggressive stimuli has no biological relevance and can cause severe tissue damage. For example, chronic inflammation is associated with carcinogenesis and obesity. Therefore, there is a need for clinical intervention [148,149,150].

The triggers of the inflammatory response involve cellular stimulation with the release of mediators, such as pro-inflammatory cytokines (i.e., TNF-α, IL-1β, IL-6, and IL-8), which promote the activation of macrophages and mast cells, that potentiate this effect by activating endothelial cells, increasing vascular permeability, leakage of fluids, proteins, and influx of immune cells (polymorphonuclear) from the circulation to the inflamed site [151]. However, this response, when uncontrolled, can lead to tissue injury.

**Figure 6:**
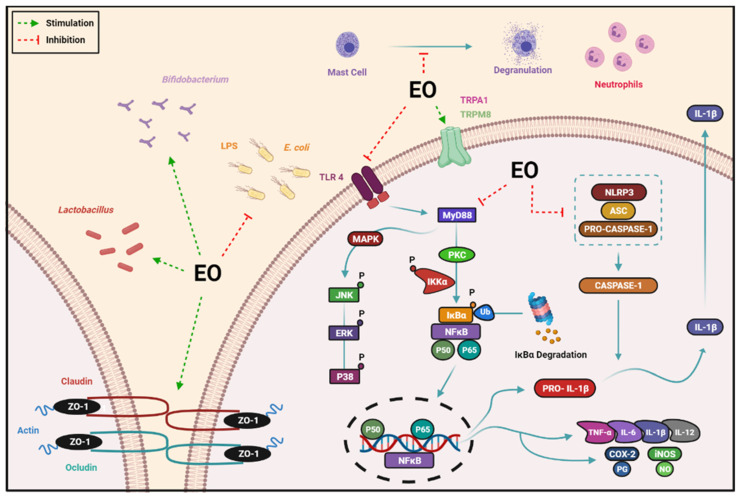
Essential oils and their main constituents modulate inflammation by reduction of inflammatory signaling pathways, activation of sensory receptors, maintenance of the epithelial barrier, and regulation of the microbiota. Note: EO = Essential oil.

In this context, several studies have revealed biological applications of essential oils in controlling the inflammatory response, based on their use in traditional medicine [152,153]. Here, we focus on the anti-inflammatory effects and mechanisms of action of essential oils and their main constituents; details can be seen in Figure 6.

### 8.2. Anti-Inflammatory Mechanisms

#### 8.2.1. Inhibition of the Release of Mediators by Inflammatory Cells

Geranium essential oil is obtained from leaves of *Pelargonium graveolens* L., a plant species of the Geraniaceae family, widely known as geranium, which is native to South Africa [154]. Geranium essential oil has been used in traditional medicine for treating inflammatory skin conditions, due to its anti-inflammatory, antiseptic, antifungal, and antibacterial potential [155,156]. Using cultured mast cells (CMC) stimulated with IgE, Kobayashi and colleagues (2016) showed that geranium essential oil reduced significantly, and dose-dependently, the β-hexosaminidase secretion, but did not inhibit its activity. This enzyme is released when mast cells, resident cells involved in the tissue allergic and inflammatory response, are activated and consequently degranulated [157]. Thus, this result suggests that geranium essential oil suppresses IgE-induced allergic response by inhibition of mast cell degranulation. The authors also characterized the chemical constituents of this essential oil and demonstrated the inhibitory effect of citronellol (54.6% inhibition), citronellyl formate (33.8% inhibition), and geraniol (32.2% inhibition) against mast cell degranulation. Additionally, citronellol inhibited IgE-induced TNF-α production in CMCs, a critical pro-inflammatory cytokine of inflammation, mediating edema, neutrophils recruitment, and activation of T cells to the inflamed site [158,159]. These results support the application of geranium essential oil and especially its major constituent, citronellol, in the treatment of allergic conditions.

α-Phellandrene is a monoterpene found in essential oils of a variety of plants, such as *Schinus molle* L. [160], *Monodora myristica* (Gaertn.) Dunal [161], and *Anethum graveolens* L. [162]. Studies have demonstrated the protective effect of α-phellandrene against ifosfamide-induced hemorrhagic cystitis [163], as well as wound healing [164], antihyperalgesic, and antidepressive properties [165]. Regarding the modulatory role on the inflammatory response, a study showed that α-phellandrene inhibited leukocyte influx and a number of rolling neutrophils or adhered to vascular endothelium, associated with carrageenan-induced acute inflammation [166]. These events of inflammation begin with the release of signaling molecules, such as cytokines and chemokines, released by resistant cells and have a fundamental role in the activation of the vascular endothelium, increasing the expression of adhesion molecules [167]. In this sense, the anti-inflammatory effect of the α-phellandrene can be explained, at least in part, by the reduction of the pro-inflammatory cytokines TNF-α and IL-6. Consistent with these results, recently, Gonçalves and collaborators (2020) reported the modulatory effect of α-phellandrene on TNF-α and IL-1β secretion using an experimental model of hemorrhagic cystitis in mice [163]. In addition, the treatment with α-phellandrene reduced the number of degranulated mast cells when mesenteric tissues were incubated with compound 48/80 (a stimulator of mast cell degranulation) [166]. Thus, these results suggest that α-phellandrene promotes mast cell stabilization, reducing the release of pro-inflammatory mediators and modulating inflammatory response.

Bergapten is a furocoumarin that consists of the main component of bergamot essential oil (*Citrus bergamia* Risso & Poit.) [168] and *Cnidium monnieri* (L.) Cuss. [169], a member of the Umbelliferae family of plants. Due to its pharmacological activity, bergapten has been used for the management of skin disorders, such as psoriasis, dermatitis, mycosis, and vitiligo. Experimentally, recent studies have reported its anti-inflammatory [170] and anti-allergic [171] effects, and its prevention of osteoporosis [172]. A possible mechanism by which bergapten controls inflammation was recently reported by Adakudugu and collaborators (2020) using an animal model of colitis. Rats treated with bergapten had a reduction in macro- and microscopic colonic lesions and colon weight-to-length ratio, an indicator of colonic edema, promoted by the damaging agent acetic acid. Associated with this, bergapten decreased the number of degranulated mast cells, compared to the acetic acid group [173]. Mast cells are strongly associated with irritable bowel disease (IBD). Barbara and collaborators (2004) demonstrated the effects of these resident cells and their degranulated state: increase in histamine and tryptase significantly increased the colonic mucosa of IBS patients [174]. Thus, it is possible that bergapten promotes mast cell granule stabilization, reducing the release of pro-inflammatory mediators, which culminates in reduced colonic damage.

#### 8.2.2. Interaction with Sensory Receptors

Carvacrol is a monoterpene phenol found in aromatic plants’ essential oils, including those of *Origanum dictamnus* L., *O. majorana* L., *Satureja montana* L., and *Thymus vulgaris* L. [175]. The biological activities of this monoterpene include antinociceptive [176], anxiolytic [177], and anti-inflammatory [178] effects. The possible interaction of carvacrol and its semisynthetic derivative, carvacryl acetate, with membrane receptors has been studied [179,180]. The administration of carvacrol or carvacryl acetate reduced 5-fluorouracil-induced intestinal mucositis, as observed by inhibition of inflammatory (i.e., TNF-α, IL-1β, KC, MPO, NF-Κb, and COX-2) and oxidative stress (i.e., GSH and MDA) markers. Additionally, the pharmacological blocker of the TRPA1 receptor with HC-030031 reversed the protective effect of carvacrol or carvacryl acetate. The TRPA1 receptor acts as a chemosensor for cell damage signals in the lumen gastrointestinal and modulates digestive functions [181], in addition to involvement in the regulation of vascular tone by the release of vasodilator neuropeptides [182]. Additionally, results of molecular docking revealed that carvacrol binds, with a strong affinity to amino acid residues, to the active site of the TRPA1 receptor [179,180]. Together, these results strongly suggest that the anti-inflammatory effects of carvacrol and carvacryl acetate are mediated, at least in part, by its interaction with the TRPA1 receptor.

Eucalyptol is a monoterpene oxide that is the major constituent of essential oils of medicinal plants such as *Eucalyptus tereticornis* Sm., *Eucalyptus globulus* Labill. [183], and *Croton rhamnifolioides* Pax & K.Hoffm. [184]. This monoterpene is used as a therapeutic alternative for respiratory diseases (i.e., asthma, bronchitis, and sinusitis) and pain management [183,185]. The anti-inflammatory effect of eucalyptol and possible action mechanism was evaluated using two animal models of inflammation: (1) Complete Freund’s Adjuvant (CFA)-induced paw inflammation and (2) Lipopolysaccharide (LPS)-induced pulmonary inflammation [186]. Using the first experimental model, the authors showed that eucalyptol strongly reduced paw edema, mechanical allodynia, and pro-inflammatory cytokines (IL-1β, TNF-α, IL-6) levels. In addition, the eucalyptol administration reduced the polymorphonuclear (PMN) infiltration, primarily neutrophils, as well as pro-inflammatory cytokines in the bronchoalveolar lavage fluid (BALF) of mice submitted to pulmonary inflammation. After elucidating the anti-inflammatory effect of eucalyptol, the authors used TRPM8^−/−^ mice to investigate the possible key role of the TRPM8 receptor in this effect. Eucalyptus is known to interact with TRP receptors, including TRPM8, where it has a reducing effect on visceral hypersensitivity [187]. In addition, studies have shown that the activation of the TRPM8 receptor can suppress the inflammatory response in colitis models [188] and during cold stress [189]. In this sense, Caceres and colleagues demonstrated that genetic deletion of the TRPM8 receptor completely abolished the anti-inflammatory effects of eucalyptol in both models and suggested that TRPM8 agonists can be a potential target for inflammatory conditions [186].

#### 8.2.3. Suppression of the NLRP3 Inflammasome

*Cinnamomum osmophloeum* Kanehira is a native plant species in Taiwan, popularly known as pseudo-cinnamomum or indigenous cinnamon, used in traditional medicine as a therapeutic alternative to treat arthritis and relieve pain and fever. Several research groups have added to the study of the biological activities of essential oils from *C. osmophloeum* and their main compounds.

In this context, Lee and collaborators (2015) studied the key mechanism involved in the control of inflammatory response promoted by essential oil from *C. osmophloeum*, linalool chemotype, which also includes cinnamaldehyde as a constituent. Using an experimental model of systemic inflammatory response syndrome (SIRS)-induced endotoxin from *Salmonella typhimurium*, the authors reported that the essential oil reduced pro-inflammatory cytokine levels, suppressed the TLR4, MD2, and MyD88 expression in mesenteric lymph nodes (MLNs) and ileum mucosa, and reversed the activation of NF-κB in both tissues. In addition, essential oil from *C. osmophloeum* suppressed NLRP3, ASC, and caspase-1 expression in MLNs and ileum [190]. During the SIRS, the endotoxin interacts with TLR4, which recruits and ligates to Myd88, a signaling cascade for activation of NF-κB and, consequently, gene overexpression of pro-inflammatory mediators. In addition, TLR4 is involved in the activation of NRPL3 inflammasome, a complex of NRLP3, ASC, and pro-caspase-1, culminating in cleavage of precursors pro-IL-1β and pro-IL-18 into mature IL-1β and IL-18 [191]. Studies reported that linalool, the main constituent of essential oil from *C. osmophloeum*, reduces the LPS-induced lung and liver inflammation [192,193]. These results strongly suggest that the protective response promoted by essential oil from *C. osmophloeum* is dependent on linalool content.

Pulegone is a monoterpenic compound, serving as the major component of essential oils from plants of *Mentha* and *Schizonepeta* genera [194]. Studies reported the potential biological activity of this monoterpene, such as antispasmodic [195], analgesic [196], and reduction of atopic dermatitis [197]. The anti-inflammatory activity of pulegone was recently reported by Yang and colleagues (2019) in an animal model of LPS-induced sepsis. Pulegone ameliorated histological changes in lung tissues and reduced the levels of IL-1β, IL-5, MIP-1β, M-CSF, and GM-CSF in the serum of septic mice. Additionally, pulegone also reduced the mRNA and protein expression of ASC, NLRP3, caspase-1, and P2X7R [198]. In another study, the modulatory effects of pulegone on the NLRP3 inflammasome signaling pathway were investigated in an in vitro model of inflammation in THP-1 cells, established using LPS + ATP or the cellular toxin nigericin. Compared to LPS + ATP/nigericin group, pulegone was able to reduce the secretion of IL-1β and IL-18, as well as ROS levels. Using molecular tools (PCR analysis and immunofluorescence), the authors demonstrated that pulegone reduced the expression of NLRP3, ASC, caspase-1, and IL-1β, as well as co-localization of the NLRP3 and ASC proteins [199]. Thus, these studies reported the ability of pulegone to regulate inflammation by mechanisms that depend, at least in part, on the suppression of the NALP inflammasome.

#### 8.2.4. Effects on NF-κB Signaling Pathway

*Citrus aurantium* L. var. *amara* Engl is a member of the family Rutaceae, widely distributed in Southeast Asia, especially in China, and used in the popular medicine and food industry. This species is known in the literature as a rich source of bioactive compounds, such as flavonoids [200], polysaccharides, [201], and essential oils [202]. Shen and colleagues (2017), using an in vitro model of LPS-stimulated RAW264.7 macrophages, showed that essential oil from *C. aurantium* suppressed the secretion of pro-inflammatory cytokines (IL-6, TNF-α, and IL-1β), NO production, and COX-2 expression. According to the authors, this potent anti-inflammatory effect promoted by essential oils is related to the inhibition of the NF-κB signaling pathway, determined by the inhibitory effect on NF-κB nuclear translocation, IκBα phosphorylation and degradation, and phosphorylation-dependent IκB kinase activation. Associated with this, the essential oil from *C. aurantium* also inhibited the phosphorylation of JNK and p38, which suggests an inhibitory effect on MAPK. The NF-κB signaling pathways play a pivotal role in regulating the expression of several genes involved in the initiation and development of the inflammatory response, such as proinflammatory cytokines, chemokines, and adhesion molecules [203]. The results also revealed that linalool (64.6 ± 0.04%), α-terpineol (7.61 ± 0.03%), (R)-limonene (6.15 ± 0.04%), and linalyl acetate (5.02 ± 0.03%) are major constituents of this essential oil [201]. In summary, these results suggest that essential oil from *C. aurantium* reduces the LPS-induced inflammatory response in RAW264.7 macrophages by suppression of MAPK/NF-κB signaling pathways, and its components can act in association or individually to induce this biological effect [201].

*Eucalyptus*, a genus of the family Myrtaceae, represents several species widely distributed in the world which are of importance economically and for human health. Among them, the species *Eucalyptus citriodora* Hook. (known as lemon-scented eucalyptus) has been reported in the literature for the extraction of essential oils and their biological potential, such as pesticidal [204], antifungal [205], analgesic, and anti-inflammatory [206]. Recently, Ho and collaborators (2020) reported the anti-inflammatory mechanism of *Eucalyptus* essential oil against LPS-stimulated RAW264.7 macrophages. The authors initially demonstrated that Eucalyptus essential oil inhibits LPS-induced NO production. Posteriorly, the essential oils were fractionated in eight subfractions (A-H), where fraction F presented high NO inhibitory activity, without affecting cell viability. Furthermore, it reduced the TNF-α and IL-6 levels, and the COX and NOS expression. Using NF-κB reporter cells (RAW Blue macrophages), the authors showed that NF-κB transcriptional activity was inhibited by fraction F in LPS-stimulated macrophages. This effect was confirmed by the suppressive effect of fraction F on phosphorylation levels of IKK-α and IκB-α. In addition, the mechanism of this anti-inflammatory effect was best elucidated by the demonstration of inhibited phosphorylation levels of JNK1/2, p38, and PKC by fraction F, which, when phosphorylated, promote downregulation of the NFkB signaling pathway, reducing the gene transcription essential to inflammatory response maintenance. In this study, the authors also elucidated the chemical composition of fraction F: methylsyringol (41.3%), 4-hydroxyl-benzenemethanol (24.4%), citronellic acid (12.7%), *trans*, *cis*-iridolactone (7.6%), menthol (4.6%), 2-phenyl ethyl anthranilate (2.2%), manool (1.8%), citronellyl anthranilate (1.1%), and citronellyltiglate (1.1%) [207]. To our knowledge, there are no reports of biological activities of the first three major compounds. Among the others, menthol is the most studied, and several studies have demonstrated its potential for regulating inflammation by inhibiting the synthesis/release of inflammatory mediators, such as IL-1β, COX-2, PGE2, and TNF-α in animal models of colitis [208], cutaneous carcinoma [209], and cystitis [210]. It was reported that essential oil extracted from *Artemisia annua* L. inhibits osteoclast differentiation. It was suggested that this action occurs via reduced TRAF6 activation and interaction on the MAPK pathway and NF-κB pathway. In addition, it probably also inhibits the expression of osteoclast-related genes. The essential oil of this plant has several volatile components, such as borneol, terpinen-4-ol, and eucalyptol. These compounds have anti-inflammatory action via different mechanisms of action [211,212]. Therefore, they can contribute to the pharmacological activity.

#### 8.2.5. Regulation of the Intestinal Microbiota and Barrier Function

Using an experimental model of colitis, Zhang and colleagues reported that essential oil of *Zanthoxylum bungeanum* Maxim. pericarp (ZBEO) ameliorated clinical features of DSS-induced colitis, such as weight loss, disease activity index (DAI), colonic edema, and histopathological alterations. Furthermore, the MPO activity (an enzyme present in granules of PMN) and pro-inflammatory cytokines (TNF-α, IL-1β, and IL-12) levels were reduced in animals treated with ZBEO. This anti-inflammatory effect promoted by ZBEO is fundamental for the regulation of colonic inflammation, since it is known that pro-inflammatory cytokines play a critical role in inflammatory bowel disease (IBD), where they promote the signaling for polymorphonuclear migration to the inflamed site, producing oxidative stress and consequent tissue damage [213]. The authors also studied the barrier function associated with colon damage and demonstrated that ZBEO recovered the expression of TJ protein, ZO-1. Maintaining the integrity of the intestinal mucosa is crucial to prevent the entry of bacteria or other harmful agents. Thus, when there is a disorganization in the rearrangement of junctional proteins, the deep layers of the intestinal wall are reached by luminous contents, leading to a disease state, both intestinal and systemic [214]. It is known that the intestinal microbiota plays a key role in the protection of the epithelium against aggressive stimuli. In this context, the authors showed that animals treated with ZBEO modulated the intestinal bacterial composition, suppressing the *E. coli* and increasing *Lactobacillus* and *Bifidobacteria* levels [215]. These results suggest that ZBOE can be an important product for the pharmaceutical and nutritional industries, due to its potential to regulate microbiota and intestinal inflammation.

Thymol is a natural monoterpene phenolic compound, serving as the main constituent of the essential oils from plants of the genera *Lippia* and *Thymus*. Studies have shown the biological activity of thymol in models of depression [216] and sciatic nerve excitability [217], as well as a potential antileishmanial agent [218]. Recently, Omonijo and collaborators, using an LPS-induced inflammation model in IPEC-J2 cells, demonstrated that thymol reduced ROS production and pro-inflammatory cytokine levels altered by LPS. These results suggest that thymol, a phenolic compound, can reduce oxidative stress due to its antioxidant effect and possible modulation of inflammatory signaling pathways (i.e., MAPK/NFkB), leading to a reduction in the inflammatory response and maintenance of cell homeostasis. Additionally, the authors demonstrated that, with a reduction in the oxidative inflammatory response related to LPS, thymol also attenuated the barrier function impairment, as demonstrated by a reduced drop in the TEER and permeability, as well as increased ZO-1 and actin. These data reflect the effect of thymol in the maintenance of epithelial barrier integrity, a pivot factor that reduces the inflammation response. On the other hand, the expression of nutrient (carbohydrates, amino acids, and proteins) transporters altered by LPS incubation had not been altered by thymol treatment [219]. Thus, monoterpenes consist of potential compounds to enhance the intestinal barrier function, associated with inflammatory response and oxidative stress, reinforcing its use as a nutritional supplement. In fact, thymol comprises 32.68% of the essential oil of *Lippia gracilis* Schauer leaves. This oil has anti-inflammatory activity [220]. In a study carried out with the essential oil of thyme (*Thymus vulgaris* L.) and its major component, thymol, both showed anti-inflammatory action, evidencing the contribution of this monoterpene in the pharmacological action of the oils that contain it in their chemical composition [221].

## 9. Conclusions

The diversity of chemical compounds found in essential oils, with components in variable amounts and proportions, explains their variability of biological and pharmacological activities. The lipophilic profile of these components contributes to their ability to penetrate cells and tissues to reach biological targets and carry out the pharmacological response. In addition, the antioxidant activity of some constituents of essential oils suggests their possible action in restoring balance in pathological disorders associated with oxidative stress, including inflammatory and tumoral processes. Conversely, the bioactivity in anti-infectious screenings shows the ease with which essential oils and their constituents cross the biological membranes of infected cells and/or microorganisms and cause their death. The evidence of these biological events makes this class of natural products a promising source in the search for new drug candidates.

## Figures and Tables

**Table 1:** Antioxidant activity of selected essential oils.

Essential Oil	Main Components ^a^	Assay	Activity	Ref.
Thyme (*T. vulgaris* L.)	Carvacrol, *p*-cymene	O_2_ uptake during inhibited autoxidation	Yes, similar to BHT	[46]
Oregano (*O. vulgare* L.)	Carvacrol, *p*-cymene	O_2_ uptake during inhibited autoxidation	Yes, similar to BHT	[46]
Savory *(Satureja hortensis* L.)	Carvacrol, *p*-cymene, γ-terpinene	O_2_ uptake during inhibited autoxidation	Yes, similar to BHT	[46]
Clove buds (*E. caryophyllus* Spreng)	Eugenol	O_2_ uptake during inhibited autoxidation	Yes, similar to BHT	[46]
Cinnamon *(C. zeylanicum* Blume)	Eugenol	O_2_ uptake during inhibited autoxidation	Yes, similar to BHT	[46]
*Melaleuca alternifolia* (*M. alternifolia* Cheel.)	Terpinen-4-ol, γ-terpinene, α-terpinene	TBARS	Yes, similar to α-tocopherol	[55]
*Cinnamomum zeylanicum* bark	Cinnamaldehyde, eugenol	β-carotene bleaching	Yes, no comparison available	[18,56]
Marine fennel (*Crithmum maritimum* L.)	γ-Terpinene, limonene,	Conjugated dienes, TBARS	Yes, similar to α-tocopherol and BHT	[57]
Common fennel (*Foeniculum vulgare* Mill.)	Estragole, α-pinene, thymol methyl ether	Conjugated dienes, TBARS	Yes, similar to α-tocopherol and BHT	[57]

(a) only components having a concentration larger than 10% are reported.

**Table 2:** Antiviral activities and mechanisms of essential oils’ constituents.

Constituent	Source	Target Virus	Mechanism	IC_50_	SI	Reference
Germacrone	*Curcuma longa* L.	Influenza	Inhibits multiple steps in the viral life cycle	6.03 μM	>41	[77]
Eugenol	*Cinnamomum zeylanicum* Blume.	Influenza	Not determined	<3.1 μL/mL	Not determined	[80]
β-Santalol	*Santalum album* L.	Influenza	Inhibits viral replication	10–100 μg/mL	Not determined	[79]
Carvacrol	*Thymus vulgaris* L.	Influenza	Not determined	2.6 μg/mL	<0.15	[76,86]
		HSV-1	Inhibits viral attachment to host cells	7 μM	43	
		Human rotavirus	Inhibits viral replication	27.9 μg/mL	33	
β-Citronellol	*Cymbopogon nardus* (L.) Rendle.	HIV-1	Inhibits reverse transcriptase	2.6 mg/mL	Not determined	[84]
β-Caryophyllene	*Cinnamomum zeylanicum* Blume. and *Syzygium aromaticum* (L.) Merrill & Perry	HSV-1	Inhibits viral attachment to host cells	0.25 μg/mL	140	[87,92,93]
		Dengue-2 virus	Inhibits multiple steps in viral life cycle	22.5 μM	71.1	
Limonene	*Citrus bergamia* Risso & Poit.	HSV-1	Inhibits viral attachment to host cells	5.9 μg/mL	10.2	[76,88]
β-Pinene	*Pinus pinaster* Aiton	HSV-1	Inhibits viral attachment to host cells	3.5 μg/mL	24.3	[88,94]
Thymol	*Thymus vulgaris* L.	HSV-1	Inhibits viral attachment to host cells	7 μM	43	[82,86]
Farnesol	*Matricaria chamomilla* L.	HSV-1	Inhibits viral attachment to host cells	3.5 μg/mL	11.4	[61,87]
*p*-Cymene	*Thymus vulgaris* L.	HSV-1	Inhibits viral attachment to host cells	>0.1%	Not determined	[89]

**Table 3:** Main mechanisms of action of essential oils and their constituents against different microorganisms.

Antimicrobial Activity	Mechanism of Action	References
Antibacterial	Plasmatic membrane and wall cell alteration.	[97]
	Change in electrical conductivity of plasma membrane.	[99]
	Decrease in ATP production.	[100]
	Change in protein synthesis.	[104]
	Inhibition of biofilm formation.	[108]
	Gene modulation.	[106]
Antifungal	Action on wall cell and plasmatic membrane.	[109]
	Inhibition of ergosterol synthesis.	[110,112]
	Decreasing intracellular ATPase activity and increasing production of Ros.	[110]
	Changes on fungal micromorphology.	[117]

**Table 4:** Some antitumor mechanisms of action have been reported for essential oils.

EO Bearing Plants	Part of the Plant Used	Main Chemical Constituents	Mechanisms Reported	References
*Euphorbia intisy* Drake	stem	heptacosane and phytol	suppression of P-gp protein/inhibition of NF-κB	[122]
*Chenopodium botrys* L.	aerial parts	α-eudesmol, elemol acetate, elemol, and α-chenopodiol-6-acetate	induction of apoptosis/augmentation of expression of p21 and p53	[132]
*Ridolfia segetum* (L.) Moris	-	α-phellandrene, terpinolene, ß-phellandrene, and dillapiol	induction of apoptosis/p21 stabilization	[133]
*Allium sativum* L.	bulb	diallyl disulfide and diallyl trisulfide	augmentation of ROS, induction of apoptosis, and differentiation	[118]
*Vitex agnus-castus* L.	leaves	1,8-cineole, eucalyptol, oleic acid, and caryophyllene	induction of apoptosis through triggering both extrinsic and intrinsic pathways	[134]
*Virola surinamensis* (Rol. ex Rottb.) Warb.	bark and leaves	aristolene, α-gurjunene, valencene, germacrene d, δ-guaiene, β-elemene, α-farnesene, bicyclogermacrene, and α-cubebene	induction of apoptotic cell death	[135]
*Cedrus deodara* (Roxb. ex D. Don) G. Don	bark	9-octadecenoic acid, copaene, and 9(*E*),11(*E*)- conjugated linoleic acid	induction of apoptosis/inhibition of NF-κB	[121]
*Conobea scoparioides* (Cham. & Schltdl.) Benth.	leaves	thymol methyl ether, thymol, and α-phellandrene	induction of apoptotic cell death	[136]
*Zataria multiflora* Boiss.	-	-	augmentation of ROS and induction of apoptosis	[119]
*Cyperus articulatus* L.	rhizome	muskatone, cyclocolorenone, α-pinene, pogostol, α-copaene, and caryophyllene oxide	induction of apoptotic cell death	[137]
*Cymbopogon citratus* (DC.) Stapf	leaves and culms	myrcene, neral and geranial	induction of apoptotic cell death	[138]
*Croton tiglium* L.	fruits	17-octadecynoic acid, tetradecanoic acid, 17-octadecynoic acid methyl ester, *n*-hexadecanoic acid, *n*-decanoic acid, linoleic acid ethyl ester, and iso-propyl 9-octadecenoate	induction of apoptotic cell death and inhibition of migration	[139]
*Zataria multiflora* Boiss.	-	carvacrol, γ-terpinene, carvacrol methyl ether, *p*-cymene, and thymol	immunomodulation	[140]
*Origanum majorana* L.	-	-	p38 MAPK-mediated protective autophagy and apoptosis	[141]
*Pinus koraiensis* Siebold & Zucc.	pinecones	α-pinene, limonene, and β-pinene	induction of apoptosis via the HIPPO/YAP signaling pathway	[142]
*Croton matourensis* Aubl.	leaves	β-caryophyllene, thunbergol, cembrene, *p*-cymene, and β-elemene	induction of apoptotic cell death	[143]
*Origanum vulgare* L.	-	thymol, *ρ*-cymene, γ-terpinene, and carvacrol	inhibition of lipogenesis and induction of apoptosis	[144]
*Aniba parviflora* (Meisn.) Mez	bark	linalool, α-humulene, δ-cadinene, α-copaene, and germacrene b	induction of apoptotic cell death	[145]
*Guatteria megalophylla* Diels	leaves	spathulenol, *γ*-muurolene, bicyclogermacrene, *β*-elemene, and *δ*-elemene	induction of apoptotic cell death	[146]
*Litsea cubeba* (Lour.) Pers.	seed	citronellal, neo-isopulegol, isopulegol, and citronellol	induction of apoptotic cell death by suppression of AKT/mTOR pathway	[120]
*Artemisia capillaris* Thunb.	-	-	induction of apoptotic cell death by activation of MAPK	[123]

## Data Availability

Not applicable.
